# Heterogenising study samples across testing time improves reproducibility of behavioural data

**DOI:** 10.1038/s41598-019-44705-2

**Published:** 2019-06-03

**Authors:** Carina Bodden, Vanessa Tabea von Kortzfleisch, Fabian Karwinkel, Sylvia Kaiser, Norbert Sachser, S. Helene Richter

**Affiliations:** 1 Department of Behavioural Biology, University of Münster, Münster, Germany; 2 Otto Creutzfeldt Center for Cognitive and Behavioral Neuroscience, University of Münster, Münster, Germany; 3 Present Address: Florey Institute of Neuroscience and Mental Health, Melbourne Brain Centre, University of Melbourne, Parkville, VIC Australia

**Keywords:** Neuroscience, Animal behaviour

## Abstract

The ongoing debate on the reproducibility crisis in the life sciences highlights the need for a rethinking of current methodologies. Since the trend towards ever more standardised experiments is at risk of causing highly idiosyncratic results, an alternative approach has been suggested to improve the robustness of findings, particularly from animal experiments. This concept, referred to as “systematic heterogenisation”, postulates increased external validity and hence, improved reproducibility by introducing variation systematically into a single experiment. However, the implementation of this concept in practice requires the identification of suitable heterogenisation factors. Here we show that the time of day at which experiments are conducted has a significant impact on the reproducibility of behavioural differences between two mouse strains, C57BL/6J and DBA/2N. Specifically, we found remarkably varying strain effects on anxiety, exploration, and learning, depending on the testing time, i.e. morning, noon or afternoon. In a follow-up simulation approach, we demonstrate that the systematic inclusion of two different testing times significantly improved reproducibility between replicate experiments. Our results emphasise the potential of time as an effective and easy-to-handle heterogenisation factor for single-laboratory studies. Its systematic variation likely improves reproducibility of research findings and hence contributes to a fundamental issue of experimental design and conduct in laboratory animal science.

## Introduction

Over the last years, a novel keyword has found its way into the scientific debate: the “reproducibility crisis”^1–4^. Current estimates assume the prevalence of irreproducible results to range between 50 and 90% in biomedical research^5,6^. Correspondingly, a recent *Nature* survey revealed that 90% of life scientists agree on the existence of a crisis of reproducibility^2^. So far, reproducibility problems have mostly been attributed to a lack of scientific rigor, methodological and statistical pitfalls as well as to current publication ethics (see refs^4,7–11^). Attempts to improve the situation, particularly in animal-based research, focused on establishing tools like the PREPARE and ARRIVE guidelines that aim to optimise planning and reporting standards and hence to reduce the risk of bias in the field^12–15^. Nonetheless, only limited progress has been made over the last years^8,16^, highlighting the need for additional improvement strategies.

Only recently, another potential source of poor reproducibility has been identified. Rigorous standardisation has long been regarded as the “gold standard” in animal research, but is currently becoming the subject of controversial debates^3^. The concept of standardisation was initially introduced to reduce variation *within* experiments and thereby to increase test sensitivity and decrease animal numbers^17,18^. At the same time, standardisation was promoted as a means of reducing variation *between* experiments in order to improve reproducibility. However, by reducing within-experiment variation, standardisation in fact limits the inference to the specific experimental conditions, resulting in statistically significant but possibly irrelevant findings (referred to as “standardisation fallacy” in the literature^3,19,20^). Increasingly rigorous standardisation within experiments therefore produces results that vary progressively between experiments, leading to poor reproducibility under real-life laboratory conditions (e.g., in behavioural phenotyping studies^21^).

Instead of neglecting all biological variation, the use of a more heterogeneous approach has been suggested to make study populations more representative^19,20,22,23^. According to the concept of “systematic heterogenisation”, the introduction of variation on a systematic and controlled basis predicts increased external validity and therefore, improved reproducibility^3,24^. Although the logic of this concept was supported by a series of proof-of-principle studies^22,23,25^, there is still no effective heterogenisation strategy for a single-laboratory experiment. Theoretically, such a strategy would require systematic variation of experimental factors within a laboratory that interact with the treatment under investigation in a way that prevents the detection of idiosyncratic or lab-specific results. With respect to preclinical animal studies, variations of, for example, batch, experimenter, or time of testing have been suggested to represent excellent starting points for a successful implementation into practice^4,10^. The aim of the present study was therefore to evaluate the potential of testing time as a feasible and easy-to-handle heterogenisation factor for single-lab studies.

For this purpose, we systematically investigated the impact of three different testing times on the reproducibility of treatment effects. More specifically, variation in treatment effects was explored by measuring behavioural differences between two of the most frequently used inbred mouse strains worldwide (C57BL/6J and DBA/2N). The phenotyping of these strains is particularly important in the context of translational research, where mice are frequently used to model human diseases. In order to reflect common practice in the field of behavioural phenotyping^26^, all mice were subjected to an established battery of five tests traditionally used to assess anxiety-like behaviour, exploratory locomotion, and spatial learning. In a subsequent approach, we used subsamples of the obtained data set and compared a standardised with a simulated heterogenised experimental design to investigate the effectiveness of testing time as a within-laboratory heterogenisation factor. We hypothesised to observe improved reproducibility of behavioural strain differences in the heterogenised compared to the standardised design.

## Results

**Figure 1:**
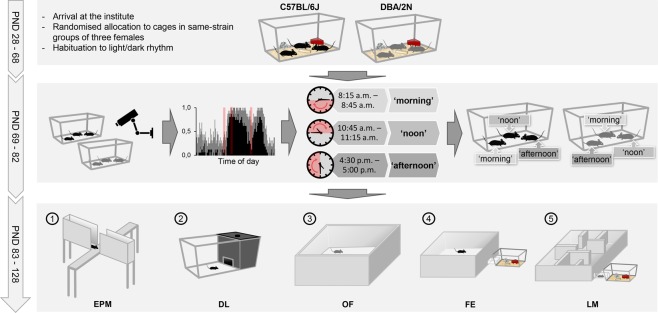
Experimental design. Female mice of the strains C57BL/6J and DBA/2N (n = 24/strain) were randomly allocated to same-strain groups of three individuals on the day of arrival (PND 28). On the basis of their 24 h home cage activity profiles, three representative time windows were chosen for behavioural testing. According to the time of day, the selected time windows were referred to as ‘morning’ (8:15 a.m.–8:45 a.m.), ‘noon’ (10:45 a.m.–11:15 a.m.), and ‘afternoon’ (4:30 p.m.–5:00 p.m.). Subsequently, each mouse per cage was assigned to a different testing group in a random way, thereby applying a classical split-plot design. All mice performed a series of behavioural tests (1–5) in the same order during the time window of the experimental group they were assigned to (‘morning’, ‘noon’, or ‘afternoon’). PND: Postnatal day, EPM: elevated plus-maze test, DL: dark-light test, OF: open-field test, FE: free-exploration test, LM: labyrinth-maze test.

Based on a comprehensive analysis of home cage activity (see Supplementary Fig. 1), we initially selected three representative time windows for conducting behavioural tests with 24 C57BL/6J and 24 DBA/2N female mice (Fig. 1; please see the Statistics section for details on the sample size calculation). Using a complex split-plot design, each of three mice within one cage was randomly allocated to one of these time windows (Fig. 1). Furthermore, we used matched pairs of C57BL/6J and DBA/2N mice that lived in neighbouring cages, thus sharing the same microenvironment. These matched pairs were always tested on the same day in the same time window (for details see Supplementary Data). Accordingly, a matched pair of mice was either tested between 8:15 a.m. and 8:45 a.m. (‘morning’ group), between 10:45 a.m. and 11:15 a.m. (‘noon’ group), or between 4:30 p.m. and 5:00 p.m. (‘afternoon’ group).

### Effects of testing time on behavioural strain differences

**Figure 2:**
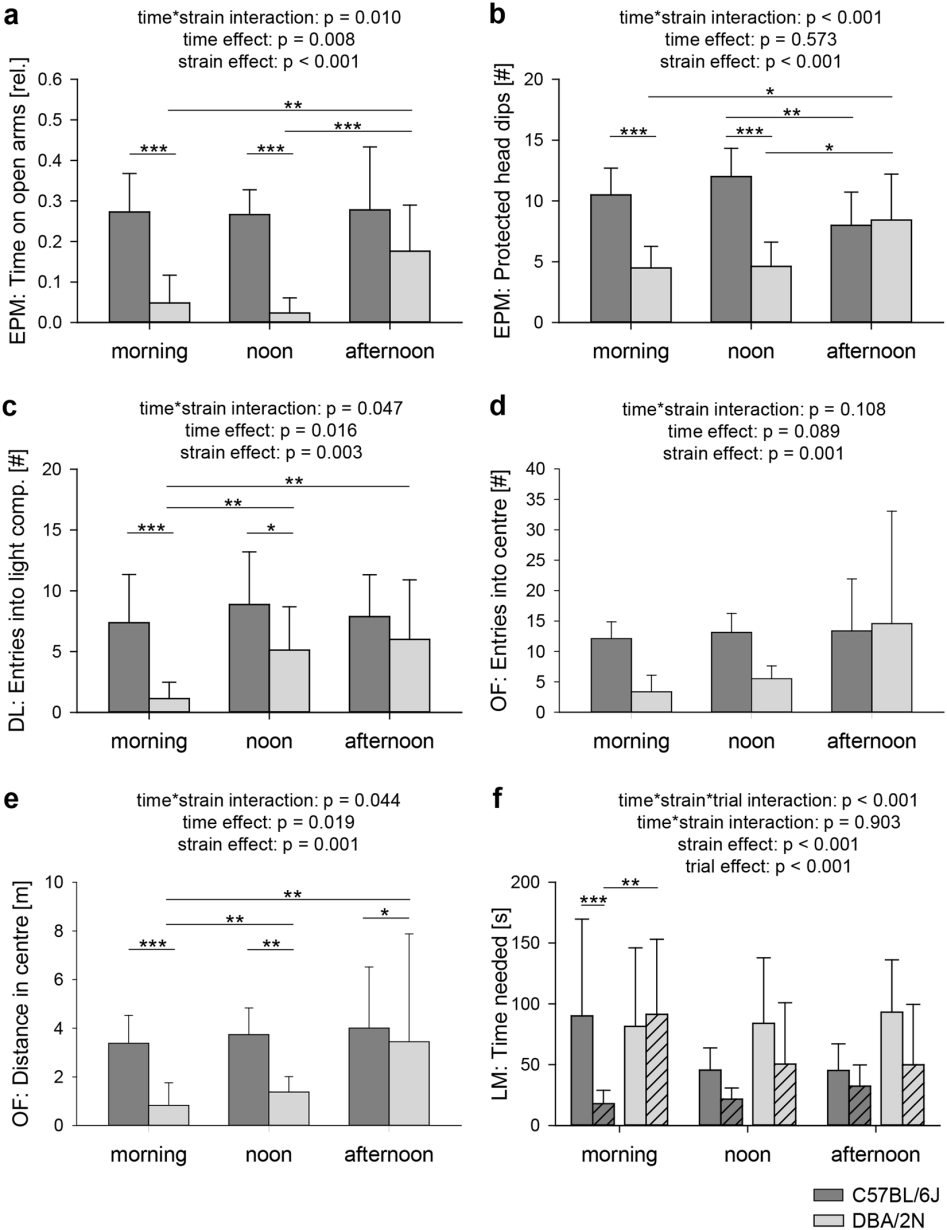
Influence of time and strain on behavioural testing. Representative selection of data of female C57BL/6J (dark grey) and DBA/2N (light grey) mice that were subjected to the elevated plus-maze (EPM), dark-light (DL), open-field (OF), and labyrinth-maze test (LM) during ‘morning’, ‘noon’, and ‘afternoon’ testing conditions, demonstrating significant interaction and main effects. Statistics: Linear mixed models, Bonferroni-Holm *post hoc* testing. Data are displayed untransformed as means + SD to ease interpretation. Every test, except for the LM, was performed only once and is represented by blank bars. With regard to the LM test, blank bars represent the 1^st^ trial, while dashed bars indicate the 2^nd^ trial after a 5-minute between-trial pause. There were significant time-by-strain interactions concerning (**a**) relative time on open arms and (**b**) protected head dips on the EPM, (**c**) number of entries into the light compartment of the DL, and (**e**) distance travelled in the centre of the OF. Furthermore, main effects of time were detected with regard to (**a**) relative time on open arms of the EPM, (**c**) number of entries into the light compartment of the DL and (**e**) centre distance in the OF. A significant main effect of strain was present with regard to all displayed parameters (**a–f**). (**f**) The time needed to reach the end of the LM was significantly influenced by a time-by-strain-by-trial interaction and trial effect. *p < 0.05; **p < 0.01; ***p < 0.001. Sample sizes: C57BL/6J: morning = 8, noon = 8, afternoon = 8; DBA/2N: morning = 8, noon = 8, afternoon = 7.

As poor reproducibility of treatment effects is statistically reflected by an interaction between an experimental or environmental factor and the treatment under investigation, we applied linear mixed models (see Methods) to screen for, in our case, significant testing time-by-strain interactions. Indeed, several significant interactions pointed towards a strong time dependence of behavioural strain differences in the EPM, DL and OF (Fig. 2; EPM: time on open arms: F_(2,27)_ = 5.441, p = 0.010, Fig. 2a; entries into open arms: F_(2,27)_ = 3.958, p = 0.031; protected head dips: F_(2,27)_ = 11.682, p < 0.001, Fig. 2b; distance on open arms: F_(2,27)_ = 7.106, p = 0.003; DL: entries into light compartment: F_(2,27)_ = 3.444, p = 0.047, Fig. 2c; OF: centre distance: F_(2,27)_ = 3.514, p = 0.044, Fig. 2e). *Post hoc* analyses revealed that in the morning and/or noon groups, DBA/2N mice showed significantly higher levels of state anxiety and less exploratory locomotion compared to C57BL/6J, whereas this difference was absent between afternoon groups (see Supplementary Table 2 for *post hoc* comparisons). Interestingly, neither the activity level in the home cage (testing at low, intermediate or high activity levels, see Methods) nor the light-dark cycle (testing during the light or the dark phase of the cycle, see Methods) seemed to be a good predictor of whether strain differences were detected or not. Furthermore, spatial learning was influenced by a significant time-by-strain-by-trial interaction (LM: number of errors: F_(2,27)_ = 7.325, p = 0.002; time to reach exit: F_(2,27)_ = 8.292, p = 0.001, Fig. 2f). As demonstrated by *post hoc* comparisons, significant differences between strains and/or trials were only found in the morning, but not in the noon and afternoon groups with respect to both the number of errors made and the time needed to solve the task (for details see Fig. 2f and Supplementary Table 3). By contrast, the two parameters assessed in the FE were not significantly influenced by such interactions (for a complete summary of statistical details see Supplementary Tables 1–4).

Though not in the focus of the present study, the analysis also revealed several significant main effects of strain, time, and/or trial on a variety of different outcome measures (see Fig. 2a–f, Supplementary Notes, and Supplementary Tables 1–3).

### Simulation approach: testing standardisation against heterogenisation

**Figure 3:**
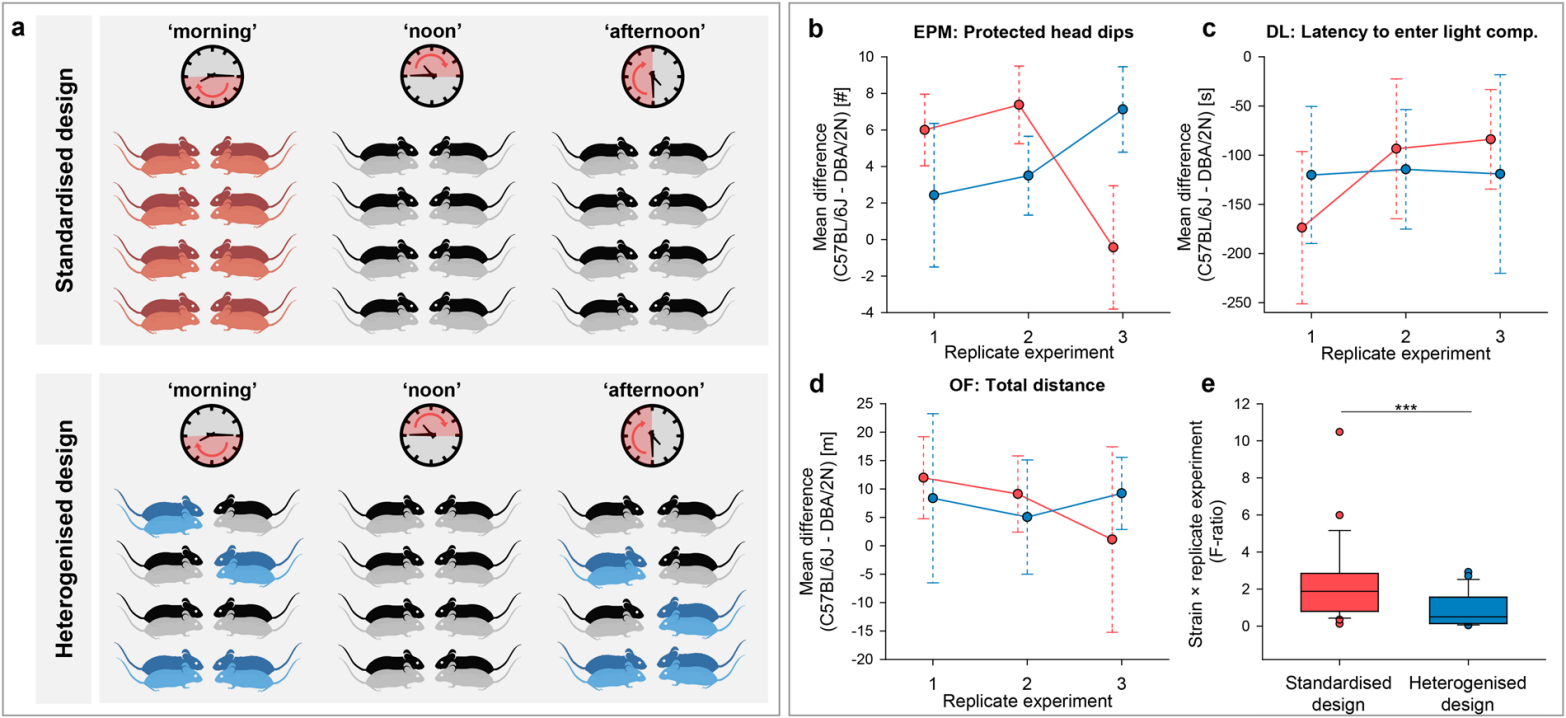
Comparison of a standardised and a heterogenised design: effects on between-replicate experiment reproducibility. (**a**) Displayed is the selection of mice to mimic one out of three standardised (red) and one out of three heterogenised replicate experiments (blue). All mice were sampled once to be part of the standardised design and once as part of the heterogenised design. In the standardised replicate experiments, all subsampled mice (n = 8) shared the same experimental background regarding the factor ‘time’. In the heterogenised design, matched pairs of C57BL/6J (dark red or dark blue mice, respectively) and DBA/2N mice (light red or light blue mice, respectively; n = 8) were randomly^39^ selected out of two different testing time groups to represent one replicate experiment. Matched pairs of mice lived in neighbouring cages, thus sharing the same microenvironment. Additionally, these mice were tested on the same day. (**b–d**) Comparisons of indicated measures from (**b**) elevated plus-maze (EPM), (**c**) dark-light (DL), and (**d**) open-field (OF) tests, showing mean strain differences (circles) with their 95% confidence intervals (dashed lines) across the three replicate experiments. (**e**) F-ratios of the ‘strain-by-replicate experiment’ interaction term were calculated separately for the two experimental designs across all 20 behavioural measures and compared using the 1-tailed Wilcoxon signed-rank test, Z = −2.912, ***p = 0.001, The analysis was based on previously published calculations^22^.

In a subsequent approach, we explored the effectiveness of testing time as a within-laboratory heterogenisation factor. For this purpose, we analysed the obtained data retrospectively to compare the reproducibility of behavioural strain differences between three standardised and three simulated heterogenised replicate experiments. Matched pairs of C57BL/6J and DBA/2N mice that originally belonged to one testing time group represented a standardised replicate experiment. In contrast, a heterogenised replicate experiment comprised a random selection of matched pairs of two different testing times (see Fig. 3a, Methods, and Supplementary data for further details on the sampling method). Mean strain differences were then determined in all behavioural measures to compare variation between three replicate experiments of the standardised against the heterogenised design. On a descriptive level, mean strain differences appeared to be more consistent among heterogenised replicate experiments than among standardised replicate experiments (Fig. 3b-d). Following previous simulations on this topic^22^, between-replicate experiment variation was statistically analysed by applying the same linear model to both designs (see Methods). F-ratios of the ‘strain-by-replicate experiment’ interaction term were then used to compare between-replicate experiment variation (i.e. reproducibility) between the two designs. Indeed, we found significantly lower values for the heterogenised compared to the standardised design (Z = −2.912, p = 0.001, Fig. 3e), indicating better reproducibility.

## Discussion

The inability to reproduce research findings is increasingly perceived as a major problem^3,24^. Although awareness has been raised and possible solutions were suggested^11^, so far, a practical implementation to improve reproducibility is still lacking^4,25^. We here demonstrate that current best practice, i.e. conducting highly standardised experiments, creates the risk for obtaining spurious and hence irreproducible results and, in a subsequent step, suggest a simple but highly effective way out of this problem.

More specifically, we show that the time of testing profoundly influenced behavioural differences between two strains of mice. In established and widely used tests for behavioural phenotyping of mouse mutants all over the world, strains were found to differ markedly in the morning and at noon, but not in the afternoon. Strikingly, poor reproducibility was particularly evident with respect to behavioural measures traditionally used to assess state anxiety and exploration^27,28^ as well as spatial learning. In contrast, measures indicative of trait anxiety^29^ remained largely unaffected by the interaction of time and strain. However, since trait anxiety is a more permanent characteristic, it might not be as easily influenced by external factors as state anxiety^29,30^, probably explaining why findings in the FE appeared to be more robust than those in the EPM, DL, and OF.

Standardisation is still a hallmark of laboratory animal science. According to relevant textbooks, this concept is widely believed as being crucial for providing valid and reproducible results. In a modelling approach, we here demonstrate that arbitrarily chosen and highly standardised time windows for testing led to idiosyncratic – in this case time-dependent – results of limited generalisability. By contrast, heterogenising the study population retrospectively across as few as two time windows significantly improved reproducibility of treatment effects between replicate experiments. Remarkably, this is in line with a recent simulation study, showing that multi-laboratory studies including not more than two laboratories produced much more consistent results than single-laboratory experiments^25^. However, multi-laboratory studies are logistically demanding and may not be appropriate for more basic or exploratory studies. Thus, the proposed idea of introducing systematic variation across testing times offers the advantage of being easily implementable within single experiments in single laboratories. Instead of conducting an experiment at one specific point in time, this would mean to split it up into at least two “mini-experiments” systematically spread over the day. Arguing from a 3R-perspective, such an approach can lead to both ‘refinement’ of animal experimentation by improving the design and conduct of experiments and ‘reduction’ by decreasing animal numbers through more meaningful single-lab experiments (see refs^24,31,32^).

In conclusion, testing time proved to play a significant role for behavioural test outcomes. Furthermore, the time of day appeared to be an effective and at the same time practically feasible heterogenisation factor. Its systematic inclusion could markedly improve external validity and thus reproducibility of behavioural data across simulated replicate experiments. Whether this approach holds true for other strains, species, and treatments, and whether it improves reproducibility of findings between laboratories remains to be tested. In light of its proof-of-principle character, however, we believe that this methodical approach would not only be useful in behavioural and cognitive neuroscience, but might also be applicable to other research branches throughout the biomedical field (e.g., for translational research).

## Methods

### Animals and housing conditions

In the present study, 24 female mice of the C57BL/6J strain as well as 24 female mice of the DBA/2N strain were used (provided by Charles River Laboratories, Research Models and Services, Germany GmbH, Sulzfeld). Both inbred strains were selected because of their widespread application in behavioural and biomedical research. The experiment was conducted in two independent batches at an interval of two weeks that were counterbalanced with respect to the experimental groups (see Experimental design). At the time of delivery, all mice were aged four weeks. They were allocated to their cages in a random way to form groups of three individuals. All mice were housed in an open cage system in standard Makrolon cages type III (floor space: 38 cm × 22 cm, height: 15 cm). Each cage contained 1.5 l softwood granules (Tierwohl, Wilhelm Reckhorn GmbH & Co. KG, Warendorf, Germany) as bedding material, a paper tissue as nesting material, and was additionally equipped with a red transparent plastic house (Mouse House™, Tecniplast Deutschland GmbH, Hohenpeißenberg, Germany) and a wooden stick. Food pellets (Altromin 1324, Altromin Spezialfutter GmbH & Co. KG, Lage, Germany) and tap water were provided *ad libitum*. Housing rooms were maintained at a 12/12 h light-dark cycle with lights off at 9:00 a.m., a temperature of about 22 °C, and a relative humidity of about 50%. In order to avoid a position bias due to variation in proximity to ventilation, lights, and human traffic, cage position was counterbalanced with respect to strain. The cages were stacked in four horizontal lines of four cages in one rack, with cages of C57BL/6J and DBA/2N mice being balanced for horizontal and vertical position in the rack, resulting in eight horizontal pairs of cages of C57BL/6J and DBA/2N mice. Mice were housed under these conditions for eight weeks before the onset of the behavioural testing phase to allow for habituation to the light-dark phase.

### Ethics statement

All procedures complied with the regulations covering animal experimentation within Germany (Animal Welfare Act) and the EU (European Communities Council DIRECTIVE 2010/63/EU) and were approved by the local (Gesundheits-und Veterinäramt Münster, Nordrhein-Westfalen) and federal authorities (Landesamt für Natur, Umwelt und Verbraucherschutz Nordrhein-Westfalen “LANUV NRW”, reference number: 84-02.04.2015.A245). The study involved behavioural testing only and did not cause distress or pain to the animals. After the study, the animals remained in the animal facility of the institute for further behavioural observations.

### Experimental design

Initially, activity levels of both C57BL/6J and DBA/2N mice were assessed in their home cages for 48 h (for a complete overview of the experimental design, see Fig. 1). Based on these observations, three experimental groups were defined in a way that reflected differences in activity levels (see Supplementary Fig. 1). Notably, the terms used to refer to the time of day are based on a human perspective. The so-called ‘morning’ group was set to the time from 8:15 a.m. to 8:45 a.m. to cover the resting phase during the light period. During the dark and thus active phase, two time windows were chosen: In the ‘noon’ group, all tests were performed between 10:45 a.m. and 11:15 a.m., while mice of the ‘afternoon’ group were subjected to all tests between 4:30 p.m. and 5:00 p.m. The allocation to the three experimental groups was based on a split-plot design, by randomly assigning one mouse per cage to a different group (see Fig. 1). These experimental designs have recently been suggested as a powerful approach to enhance statistical power of small-scale studies, allowing fewer animals to be used and contributing to increased reproducibility^33^.

All behavioural tests were performed between postnatal day (PND) 83 and 128. Due to the death of one mouse before the start of behavioural testing, the sample size of one group (‘afternoon’ group of the DBA/2N strain) was reduced to n = 7. At each testing time, matched pairs of C57BL/6J and DBA/2N mice out of neighbouring cages were subjected to behavioural testing. The order of testing the animals within one testing session was randomised. Furthermore, only one individual per cage was tested on any given day. Between two tests for the same mouse, there was a pause of at least one day. This procedure ought to minimise possible disturbances in the activity profile.

### Behavioural testing

In order to assess anxiety-like behaviour, exploratory locomotion, and spatial learning, the elevated plus-maze (EPM), dark-light (DL), open-field (OF), free-exploration (FE), and labyrinth-maze tests (LM) were performed. Parameters assessed in the EPM, DL, and OF are assumed to reflect state anxiety and exploratory locomotion^27,28^, while FE measures are indicative of trait anxiety^29,34^. LM parameters are thought to demonstrate spatial learning abilities. Each test was conducted under dim light conditions in a testing room a few meters away from the housing room. During the transport, the cage was protected from light. The order of tests was the same for each subject. Between subjects, each apparatus was thoroughly cleaned with 70% ethanol and dried afterwards. The animal’s movements were recorded by a webcam (Webcam Pro 9000, Logitech, Europe S.A., Lausanne, Switzerland) and automatically analysed by the video tracking system ANY-maze (Version 4.99, Stoelting Co., Wood Dale, USA). Due to different fur colours of mice (C57BL/6 mice: black, DBA/2N mice: brown) and clearly defined testing times, blinding was not possible at this stage of the experiment. However, since the aim of the present study was to investigate the reproducibility of treatment (i.e. strain) effects across different testing times, the presence or absence of blinding procedures was unlikely to influence the main outcome (i.e. reproducibility).

### Elevated plus-maze test

The EPM was performed on PND 88 ± 5. The plus-shaped apparatus consisted of two opposing open arms (30 cm × 5 cm), two opposing closed arms (30 cm × 5 cm), and a central square (5 cm × 5 cm). While the closed arms were equipped with 20 cm high walls, the open arms were surrounded by only a small lip (4 mm) that prevented mice from falling off. The EPM was elevated 50 cm above the ground. Illumination intensity in the centre square was 25 lx. After spending 1 min in an empty box protected from light, each mouse was individually placed on the central platform facing a closed arm and was allowed to freely explore the apparatus for 5 min. The parameters measured were the relative time spent on the open arms (*time on open arms / (time on open* + *time on closed arms)*), the relative entries into the open arms (*entries into open arms / (entries into open* + *entries into closed arms)*), and the relative distance travelled on the open arms (*distance on open arms / (distance on open* + *distance on closed arms*) to assess anxiety-like behaviour. The sum of entries into the open and closed arms, the total distance as well as the number of protected head dips (*mouse lowers its head over the side of an open arm with its ears protruding over the edge, while at least the hind limbs remain in the closed segment or central platform*) were assessed as indicators of exploratory locomotion.

### Dark-light test

The DL was performed at the age of 94 ± 4 using a modified Makrolon cage type III, which was separated into two compartments by a dark plastic panel comprising a sliding door. The dark compartment (17 cm × 27 cm × 15 cm) made up approximately one third of the cage, had opaque walls and an opaque lid and was unlit, while the light compartment (26 cm × 27 cm × 15 cm) had transparent walls and no lid. Illumination (40 lx) was provided by an LED lamp. Each mouse was placed inside the dark compartment with the lid and sliding door closed and remained there for 1 min before the sliding door was opened and the mouse was allowed to freely explore the apparatus for 5 min. The parameters analysed were the latency to enter and the time spent in the light compartment as indicators of anxiety-like behaviour, and the number of entries into the light compartment to assess exploratory locomotion.

### Open-field test

The OF was conducted at the age of 101 ± 3 days. The apparatus consisted of a square arena (80 cm × 80 cm) surrounded by walls (42 cm) and made out of white coated plywood. The illumination level was set to 35 lx. After spending 1 min in an empty box protected from light, each mouse was individually placed in one corner of the OF facing the wall and was allowed to freely explore the arena for 5 min. The parameters analysed were the time spent in the centre of the arena (defined as the area of the OF being located at least 20 cm distant from the walls), the number of entries into the centre, and the distance travelled in the centre to measure anxiety-like behaviour. The total distance travelled in the OF was assessed as a measure for exploratory locomotion.

### Free-exploration test

At the age of 109 ± 5 days, the mice were tested in the FE. The apparatus consisted of a square arena (60 cm × 60 cm) surrounded by walls (36 cm) and made of white coated plywood. An LED lamp set to 35 lx was mounted above the testing apparatus to provide illumination. The arena was connected to the home cage of the mouse via a sliding door and a transparent plastic tunnel (24 cm × 15 cm × 10 cm). Prior to testing, the cage mates of the test subject were removed from the home cage and placed in another cage containing a small amount of bedding material from the home cage, a red house, a paper tissue, and food and water *ad libitum*. After spending 1 min in an empty box protected from light, the mouse was placed in its home cage, with the sliding door open, allowing the animal to freely explore the FE for 15 min. The parameters measured were the latency to enter the arena, the number of excursions into the arena, and the time spent in the arena. After the test, the cage mates of the test subject were placed back into the home cage and the cage was left undisturbed for at least one day before the next animal of the cage was tested.

### Labyrinth-maze test

The labyrinth-maze (LM) was performed on PND 121 ± 8. The apparatus consisted of a white platform (40 cm × 24 cm) with several transparent acrylic glass walls (15 cm), partly with passageways to form a labyrinth. Altogether, there were 7 passageways, with only a restricted number leading to the home cage, which was connected via a short tunnel (8 cm). Before testing, the cage mates of the test subject were placed in another cage for the time of testing. The empty home cage was connected to the end of the LM while the test subject was placed in an empty box protected from light for 1 min prior to testing. Thereafter, it was placed in the start position of the LM, allowing it to freely explore the apparatus and find its way to the home cage within 5 min. After having solved the task by reaching the home cage, the mouse had a 5 min pause in its home cage, while the LM was thoroughly cleaned with 70% ethanol. Subsequently, the mouse was again placed in the start position to perform a second trial for 5 min maximum. After the second test, the cage mates of the test subject were placed back into the home cage and the cage was left undisturbed for at least 1 day before the next animal of the cage was tested. The parameters measured were the time needed to exit the LM and the number of errors, meaning all transits through passageways that did not lead to the exit.

### Statistics

All parameters were analysed using linear mixed models. To meet the assumptions of parametric analysis, residuals were graphically examined for normal distribution, homoscedasticity and outliers, and the Shapiro-Wilk test was applied. When necessary, raw data were transformed using square root, logarithmic, inverse or angular transformations (see Supplementary Table 1). Specifically, linear mixed models were used to analyse dependent variables with fixed between-subject factors ‘time’ and ‘strain’, and the random between-subject factor ‘cage’ nested within ‘strain’. Likewise, linear mixed models for repeated measures were performed for the analysis of spatial learning with fixed within-subjects factor ‘trial’, fixed between-subject factors ‘time’ and ‘strain’, the interaction of ‘time’ and ‘strain’ and ‘trial’, and the random between-subjects factor ‘cage’ nested within ‘strain’. Main effects and interaction terms were tested on local significance level alpha (α) = 0.05. If there were significant main or interaction effects, *post hoc* pairwise comparisons of different factor levels were conducted using Bonferroni-Holm adjustments. To present the magnitude of the reported effects in a standardised metric, effect sizes were calculated as partial eta squared (η2 p; ref.^35^). Raw data were summarised as means with standard deviations in Supplementary Table S4.

A statistical power analysis was performed for sample size estimation. Taking into account all parameters that yielded large effect sizes, we could ensure that a total sample size of 48 mice (n = 8 per group) could detect biologically relevant differences with a power of 80%^36^. All statistical analyses were conducted using the statistical software IBM SPSS Statistics (IBM Version 23, Release 2015), R^37^ or G*Power^38^. Graphs were created using the software SigmaPlot 12.5 for Windows (Build 12.5.0.38, Systat Software, Inc. 2011).

### Simulation approach: testing standardisation against heterogenisation

In the second part of the study, we analysed the present data retrospectively, comparing the reproducibility of behavioural strain differences of three standardised to three simulated heterogenised replicate experiments. For this comparison, the data of all animals were used once to mimic a standardised design and once to simulate a heterogenised design. Notably, heterogenising study populations does not mean to introduce variation in a random and hence uncontrolled way. Rather, variation needs to be systematically included in a way that prevents reduced test sensitivity or statistical power^32,33^. Accordingly, those C57BL/6J mice (n = 8) and DBA/2N mice (n = 8) that originally belonged to one testing time group, e.g. ‘morning’, represented one standardised replicate experiment (see Fig. 3a, standardised design). In contrast, a heterogenised replicate experiment comprised a random^39^ selection of C57BL/6J mice (n = 8) and matched DBA/2N mice (n = 8) out of two different testing times (see Fig. 3a, heterogenised design; Supplementary Fig. 2 and Supplementary Data). Mice were thus chosen to either decrease (‘standardised’ replicate experiments) or increase (‘heterogenised’ replicate experiments) experimental variation within replicate experiments. The computer-based randomised subsampling procedure allowed for an unbiased allocation of mice to the different designs and formed the basis for the subsequent analysis. We determined mean strain differences (*mean C57BL/6J mice* – *mean DBA/*2*N mice*) with their 95% confidence intervals for all 20 behavioural measures to descriptively compare variation among the three replicate experiments for the standardised and the heterogenised design. The between-replicate experiment variation was statistically analysed by applying the same linear model with ‘strain’, ‘replicate experiment’, and the interaction of ‘strain’ and ‘replicate experiment’ as fixed factors to both designs. Notably, the term ‘replicate experiment’ in the standardised design was identical to the ‘testing time’ term of the previous analysis, whereas it reflected the three simulated experiments in the heterogenised design. F-ratios for the 20 behavioural measures of the ‘strain-by-replicate experiment’ interaction term were calculated for each experimental design (standardisation versus heterogenisation) and compared using the 1-tailed Wilcoxon signed-rank test. The analysis was based on previous simulations on this topic^22^ (but see also discussions about how to approach reproducibility from a statistical perspective^11,40,41^).

## Supplementary information

Supplementary information

## Data Availability

The datasets generated and/or analysed during the current study are available in the Figshare repositories. https://figshare.com/s/95df5b769d9db87bfbe5 and https://figshare.com/s/f9369621c90bd46a96b8 and https://figshare.com/s/63a7564621645d96f87f and https://figshare.com/s/7d3ae57105aa5b0c2468.
