# Supplementary material for: Heterogenising study samples across testing time improves reproducibility of behavioural data
Source: Sci Rep. 2019 Jun 3;9:8247. doi: 10.1038/s41598-019-44705-2 (PMC6547843; doi:10.1038/s41598-019-44705-2)
Supplement: Supplementary file 1 — Supplementary information [file 41598_2019_44705_MOESM1_ESM.pdf]

Heterogenising study samples across testing time improves reproducibility of behavioural data

Carina Bodden[1,3], Vanessa Tabea von Kortzfleisch[1,2], Fabian Karwinkel[1], Sylvia Kaiser[1,2], Norbert Sachser[1,2] & S. Helene Richter[1,2*]

[1] Department of Behavioural Biology, University of Münster, Münster, Germany

[2] Otto Creutzfeldt Center for Cognitive and Behavioral Neuroscience, University of Münster, Münster, Germany

[3] Present Address: Florey Institute of Neuroscience and Mental Health, Melbourne Brain Centre, University of Melbourne, Parkville, VIC, Australia

Correspondence and requests for materials should be addressed to S.H.R. (email: richterh@uni-muenster.de)

Supplementary Information

Supplementary Notes

Though not in the focus of this paper, linear mixed model analyses revealed the following significant main effects:

Main effects of time: There was a significant main effect of time on behavioural parameters assessed in the EPM, DL, and OF (**EPM**: relative time on open arms: $F_{(2,27)} = 5.803$, $p = 0.008$, Fig. 2a; entries into open arms: $F_{(2,27)} = 7.072$, $p = 0.003$; open arm distance: $F_{(2,27)} = 10.260$, $p < 0.001$; **DL**: latency to enter light compartment: $F_{(2,27)} = 8.492$, $p = 0.001$, number of entries into light compartment: $F_{(2,27)} = 5.580$, $p = 0.009$, Fig. 2c; time spent in light compartment: $F_{(2,27)} = 5.399$, $p = 0.011$; **OF**: centre distance: $F_{(2,27)} = 4.638$, $p = 0.019$, Fig. 2e). *Post hoc* testing demonstrated that levels of state anxiety were significantly lower in the afternoon compared to both the morning and noon groups. Additionally, state anxiety was lower in the noon compared to the morning groups. Exploratory locomotion was increased in the noon and afternoon groups in comparison with the morning groups (for *post hoc* comparisons see Supplementary Table 2).

Main effects of strain: A significant main effect of strain was detected concerning EPM, DL, OF, and LM measures. Specifically, DBA/2N mice displayed higher levels of state anxiety and lower levels of exploration compared to C57BL/6J mice (**EPM**: less time on open arms: $F_{(1,14.387)} = 68.583$, $p < 0.001$, Fig. 2a; fewer entries into open arms: $F_{(1,14.224)} = 40.135$, $p < 0.001$; shorter distances on open arms: $F_{(1,14.426)} = 78.453$, $p < 0.001$; less protected head dips: $F_{(1,14.229)} = 28.038$, $p < 0.001$, Fig. 2b; shorter total distance: $F_{(1,14.284)} = 35.529$, $p < 0.001$; **DL**: greater latency to enter light compartment: $F_{(1,14.111)} = 31.164$, $p < 0.001$; fewer entries into light compartment: $F_{(1,14.204)} = 15.491$, $p = 0.001$, Fig. 2c; less time in light compartment: $F_{(1,14.203)} = 16.539$, $p = 0.001$; **OF**: less centre entries: $F_{(1,14.242} = 18.729$, $p = 0.001$, Fig. 2d; less centre time: $F_{(1,14.244)} = 11.900$, $p = 0.004$; shorter centre distance: $F_{(1,14.114)} = 17.467$, $p = 0.001$, Fig. 2e). Furthermore, DBA/2N mice showed impaired spatial learning abilities compared to C57BL/6J mice (**LM**: more time to find exit: $F_{(2,27)} = 12.004$, $p = 0.001$, Fig. 2f).

Main effects of trial: There was a significant trial effect regarding the LM, demonstrating improvements from the first to the second trial (**LM**: less errors: $F_{(2,27)} = 22.548$, $p < 0.001$; less time needed: $F_{(2,27)} = 40.965$, $p < 0.001$, Fig. 2f).

Supplementary Figures

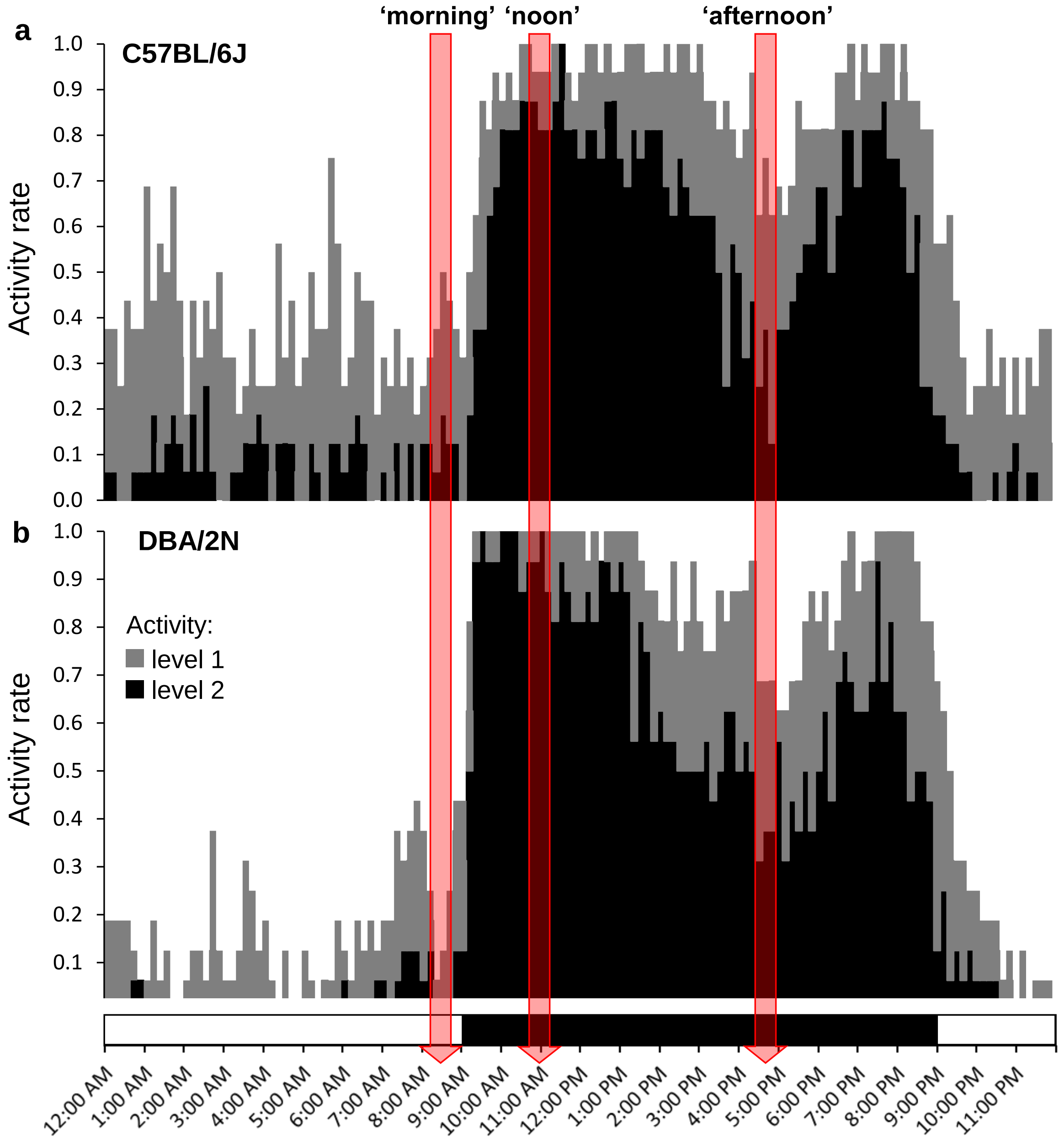

Supplementary Figure 1. Activity profile of female C57BL/6J and DBA/2N mice. Home cage activity per cage was assessed prior to behavioural testing using video-recordings. Video recordings took place between PND 70 and 74 for 48 h hours over the course of the light and dark phase. For this purpose, infrared lamps were used and surveillance cameras sensitive to infrared wavelengths (EH1000H-4 Nano cameras, AVer Information Inc., Taiwan) were mounted approximately 50 cm above the cages. At an interval of 10 min, the activity level within each cage was assessed using instantaneous sampling[40]. The activity was rated as level 0 if all mice in one cage were inactive, meaning they were lying or sitting motionlessly (except for tiny whisker, ear, or tail movements). Whenever there was minor activity in terms of small

movements within the nest or only one mouse moving around, level 1 applied. In case of high activity, with at least two mice moving in the cage, the activity was considered level 2 (definitions of active and inactive are based on previous publications[41,42]). The order in which mice were observed was pseudo-randomised, meaning that mice of different strains and different batches were recorded and observed alternatingly. Illustrated is the relative frequency of each activity level at each sampling point for 24 h, arithmetically averaged from data for 48 h for mice of the strain (**a**) C57BL/6J and (**b**) DBA/2N. Red boxes indicate the testing times 'morning', 'noon', and 'afternoon', with each time window lasting 30 min. The horizontal white/black bar represents the reversed 12/12 h light-dark cycle, with lights off at 9 a.m. Please note that the terms used to refer to the time of day are based on a human perspective. Sample size: 8 cages (à 3 mice) per strain.

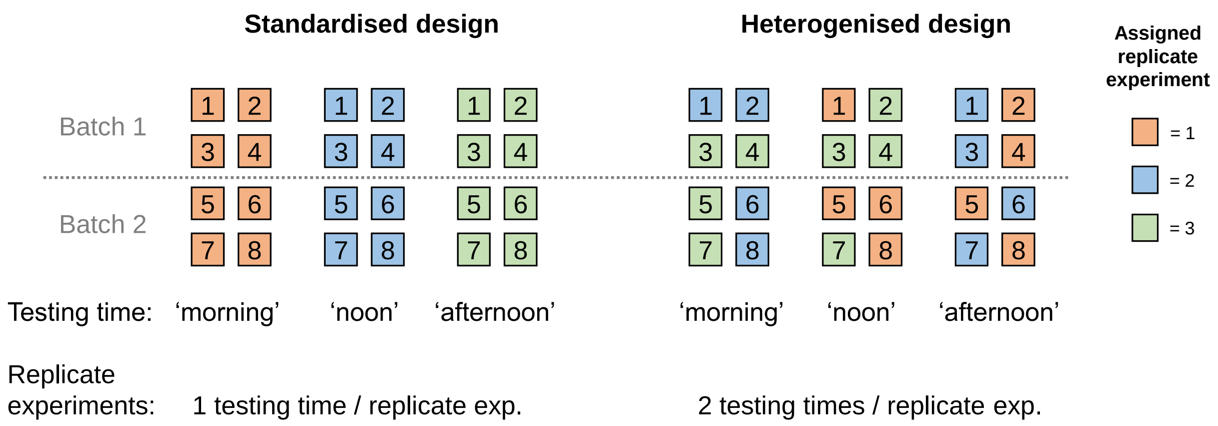

Supplementary Figure 2. Subsampling procedure for the standardised and simulated heterogenised design. Each animal was sampled once in each design and assigned to one replicate experiment of the respective design. In the standardised design, for example, all 8 animals tested at time point 'morning' were assigned to the standardised replicate experiment 1. In contrast, each heterogenised replicate experiment comprised data of two randomly selected testing times, for example replicate experiment 1 (orange) comprised data from testing times 'noon' and 'afternoon'. 4 out of 8 animals of the 'noon' (1, 5, 6, 8) and 'afternoon' groups (2, 4, 5, 8) were randomly selected to become part of the heterogenised replicate experiment 1.

Supplementary Tables

Supplementary Table 1. Statistical details on the linear mixed models. Presented are interaction and main effects of 'time' and 'strain' (F-ratios, p-values, and estimated effect sizes) on individual common parameters that were assessed in the elevated plus-maze (EPM), dark-light (DL), open-field (OF) and free-exploration tests (FE). Furthermore, main and interaction effects of 'trial' are presented for two successive trials in the labyrinth-maze test (LM). Data were transformed (Transf.) whenever deviating from normal distribution. ang = angular, lg = logarithmic, sqrt = square root, inv = inverse transformation. P-values in bold represent statistically significant differences ($p < 0.05$). Partial eta squared (η2 p) values are presented as effect size measures. For details on statistical analyses, see Methods. state anx. = state anxiety; expl. = exploration; trait anx. = trait anxiety.

Supplementary Table 2. Statistical details on *post hoc* comparisons of EPM, DL, and OF parameters. Presented are p-values for within-group or between-group comparisons, respectively, regarding common parameters that were assessed in the elevated plus-maze (EPM), dark-light (DL), and open-field tests (OF) and yielded significant differences. Data were transformed whenever deviating from normal distribution. P-values in bold represent statistically significant differences ($p < 0.05$). For details on statistics, see Methods.

Supplementary Table 3. Statistical details on *post hoc* comparisons of LM parameters. Presented are p-values for within-group or between-group comparisons, respectively, regarding parameters that were assessed in the labyrinth-maze test (LM). Data were transformed whenever deviating from normal distribution. P-values in bold represent statistically significant differences ($p < 0.05$). For details on statistics, see Methods.

Supplementary Table 4. Behavioural data analysis. Presented are the means and standard deviations (SD) of common parameters, assessed in the elevated plus-maze (EPM), dark-light (DL), open-field (OF), free-exploration (FE), and labyrinth-maze test (LM) that were displayed by female C57BL/6J and DBA/2N mice during 'morning', 'noon', or 'afternoon' testing conditions.

Supplementary Table 1

Test	Parameter	Domain	Transf.	time*strain			time			strain		
				F-ratio	p-value	η2 p	F-ratio	p-value	η2 p	F-ratio	p-value	η2 p
EPM	**Relative open arm time**	state anx.	ang	5.441	**0.010**	0.287	5.803	**0.008**	0.301	68.583	**< 0.001**	0.827
	**Relative open arm entries**	state anx.	-	3.958	**0.031**	0.227	7.072	**0.003**	0.344	40.135	**< 0.001**	0.738
	**Relative open arm distance**	state anx.	ang	7.106	**0.003**	0.345	10.260	**< 0.001**	0.432	78.453	**< 0.001**	0.845
	**Protected head dips [#]**	expl.	-	11.682	**< 0.001**	0.464	0.569	0.573	0.040	28.038	**< 0.001**	0.663
	**Total distance [m]**	expl.	lg	1.338	0.279	0.090	2.016	0.153	0.130	35.529	**< 0.001**	0.713
	**Sum of entries [#]**	expl.	lg	0.140	0.870	0.010	0.536	0.591	0.038	4.138	0.061	0.222
DL	**Time in light comp. [s]**	state anx.	sqrt	1.843	0.178	0.120	5.399	**0.011**	0.286	16.539	**0.001**	0.538
	**Entries into light comp. [#]**	expl.	sqrt	3.444	**0.047**	0.203	5.580	**0.009**	0.292	15.491	**0.001**	0.522
	**Latency to enter light comp. [s]**	state anx.	lg	0.632	0.539	0.045	8.492	**0.001**	0.386	31.164	**< 0.001**	0.688
OF	**Center Time [s]**	state anx.	sqrt	0.506	0.608	0.036	2.798	0.079	0.172	11.900	**0.004**	0.455
	**Center entries [#]**	state anx.	lg	2.420	0.108	0.152	2.644	0.089	0.164	18.729	**0.001**	0.568
	**Center distance [m]**	state anx.	inv	3.514	**0.044**	0.207	4.638	**0.019**	0.256	17.467	**0.001**	0.553
	**Total distance [m]**	expl.	lg	0.896	0.420	0.062	0.819	0.451	0.057	14.030	**0.002**	0.492
FE	**Time in arena [s]**	trait anx.	-	1.302	0.289	0.088	2.136	0.138	0.137	0.549	0.471	0.037
	**Entries into arena [#]**	trait anx.	-	2.202	0.130	0.140	2.202	0.130	0.140	0.001	0.982	0.001
	**Latency to enter arena [s]**	trait anx.	lg	1.713	0.199	0.113	0.972	0.391	0.067	0.750	0.401	0.050
LM	**Time needed [s]**	learning	lg	0.102	0.903	0.002	0.521	0.5978	0.012	12.004	**0.001**	0.226
	**Errors made [#]**	learning	lg	0.321	0.727	0.008	0.232	0.7940	0.006	0.185	0.669	0.004

Test	Parameter	Domain	Transf.	time*strain*trial [1]			time*trial [1]			strain*trial [1]			trial [1]		
				F-ratio	p-value	η2 p	F-ratio	p-value	η2 p	F-ratio	p-value	η2 p	F-ratio	p-value	η2 p
LM	**Time needed [s]**	learning	lg	8.292	**0.001**	0.144	0.103	0.902	0.003	2.920	0.095	0.066	40.965	**< 0.001**	0.500
	**Errors made [#]**	learning	lg	7.325	**0.002**	0.132	1.497	0.236	0.034	2.785	0.103	0.064	22.548	**< 0.001**	0.355

[1] Data derived from mixed model ANOVA with repeated measures which was only performed for parameters assessed in the LM.

Supplementary Table 2

Test	Parameter	C57BL/6J morning vs noon	C57BL/6J morning vs afternoon	C57BL/6J noon vs afternoon	DBA/2N morning vs noon	DBA/2N morning vs afternoon	DBA/2N noon vs afternoon	C57BL/6J vs DBA/2N morning	C57BL/6J vs DBA/2N noon	C57BL/6J vs DBA/2N afternoon	Overall time comparisons morning vs noon	Overall time comparisons morning vs afternoon	Overall time comparisons noon vs afternoon
EPM	Relative open arm time	1.000	1.000	1.000	1.000	**0.003**	**0.001**	**< 0.001**	**< 0.001**	0.142	1.000	**0.032**	**0.012**
	Relative open arm entries	1.000	1.000	1.000	0.092	**0.002**	**0.001**	**< 0.001**	**< 0.001**	0.068	1.000	**0.011**	**0.007**
	Relative open arm distance	1.000	1.000	1.000	1.000	**< 0.001**	**< 0.001**	**< 0.001**	**< 0.001**	0.236	1.000	**0.005**	**0.001**
	Protected head dips [#]	0.653	0.135	**0.007**	1.000	**0.011**	**0.014**	**< 0.001**	**< 0.001**	0.698			
DL	Time in light comp. [s]										**0.037**	**0.019**	1.000
	Entries into light comp. [#]	1.000	1.000	1.000	**0.005**	**0.003**	1.000	**< 0.001**	**0.040**	0.194	**0.022**	**0.024**	1.000
	Latency to enter light comp. [s]										**0.001**	0.376	0.070
OF	Center distance [m]	1.000	1.000	1.000	**0.009**	**0.004**	1.000	**< 0.001**	**0.003**	**0.019**	**0.052**	**0.035**	1.000

Supplementary Table 3

**C57BL/6J:**	**Within-group comparisons**			**Between-group comparisons**					
	**trial 1 vs. 2**			**morning vs. noon**		**noon vs. afternoon**		**morning vs. afternoon**	
**Parameter**	**morning**	**noon**	**afternoon**	**trial 1**	**trial 2**	**trial 1**	**trial 2**	**trial 1**	**trial 2**
**Time needed**	**<0.001**	0.357	1.000	1.000	1.000	1.000	1.000	1.000	1.000
**Errors made**	**<0.001**	1.000	1.000	1.000	1.000	1.000	1.000	1.000	1.000

**DBA/2N:**	**Within-group comparisons**			**Between-group comparisons**					
	**trial 1 vs. 2**			**morning vs. noon**		**noon vs. afternoon**		**morning vs. afternoon**	
**Parameter**	**morning**	**noon**	**afternoon**	**trial 1**	**trial 2**	**trial 1**	**trial 2**	**trial 1**	**trial 2**
**Time needed**	1.000	0.317	0.208	1.000	1.000	1.000	1.000	1.000	1.000
**Errors made**	1.000	1.000	1.000	1.000	1.000	1.000	1.000	1.000	1.000

**C57BL/6J vs. DBA/2N:**	**Between-group comparisons**					
	**morning**		**noon**		**afternoon**	
**Parameter**	**trial 1**	**trial 2**	**trial 1**	**trial 2**	**trial 1**	**trial 2**
**Time needed**	1.000	**0.003**	1.000	1.000	1.000	1.000
**Errors made**	1.000	1.000	1.000	1.000	1.000	1.000

Supplementary Table 4

Test	Parameter	C57BL/6J morning Mean ± SD	C57BL/6J noon Mean ± SD	C57BL/6J afternoon Mean ± SD	DBA/2N morning Mean ± SD	DBA/2N noon Mean ± SD	DBA/2N afternoon Mean ± SD
EPM	Relative open arm time	0.27 ± 0.10	0.27 ± 0.06	0.28 ± 0.16	0.05 ± 0.07	0.02 ± 0.04	0.18 ± 0.11
	Relative open arm entries	0.37 ± 0.11	0.37 ± 0.09	0.40 ± 0.12	0.10 ± 0.11	0.09 ± 0.06	0.28 ± 0.12
	Relative open arm distance	0.27 ± 0.08	0.23 ± 0.04	0.28 ± 0.09	0.04 ± 0.07	0.02 ± 0.05	0.20 ± 0.12
	Protected head dips [#]	10.50 ± 2.20	12.00 ± 2.33	8.00 ± 2.73	4.50 ± 1.77	4.63 ± 2.00	8.43 ± 3.78
	Total distance [m]	10.49 ± 2.33	11.31 ± 2.22	10.87 ± 2.65	6.11 ± 1.26	7.03 ± 1.50	8.51 ± 1.80
	Sum of entries [#]	24.88 ± 6.40	25.63 ± 6.14	26.88 ± 8.66	21.00 ± 8.00	22.88 ± 6.13	23.00 ± 4.86
DL	Time in light comp. [s]	54.25 ± 35.20	76.00 ± 46.75	67.38 ± 40.33	7.63 ± 9.10	30.38 ± 22.63	43.71 ± 32.80
	Entries into light comp. [#]	7.38 ± 3.96	8.88 ± 4.32	7.88 ± 3.44	1.13 ± 1.36	5.13 ± 3.56	6.00 ± 4.90
	Latency to enter light comp. [s]	44.00 ± 68.59	9.75 ± 6.54	30.25 ± 40.16	217.75 ± 88.25	103.25 ± 102.35	114.14 ± 56.98
OF	Center Time [s]	15.99 ± 5.40	19.15 ± 3.08	21.05 ± 10.65	8.08 ± 8.96	9.61 ± 2.89	14.93 ± 13.74
	Center entries [#]	12.13 ± 2.75	13.13 ± 3.14	13.38 ± 8.55	3.38 ± 2.72	5.50 ± 2.14	14.57 ± 18.49
	Center distance [m]	3.38 ± 1.15	3.74 ± 1.10	4.00 ± 2.51	0.83 ± 0.93	1.38 ± 0.63	3.45 ± 4.43
	Total distance [m]	34.98 ± 8.16	36.46 ± 7.55	34.55 ± 10.38	23.01 ± 6.47	27.36 ± 6.07	33.46 ± 19.78
FE	Time in arena [s]	198.31 ± 162.69	103.02 ± 122.54	202.09 ± 128.13	116.31 ± 69.03	126.90 ± 80.48	166.91 ± 87.12
	Entries into arena [#]	13.75 ± 9.77	10.50 ± 12.14	21.25 ± 10.36	6.88 ± 3.91	11.00 ± 6.14	16.00 ± 12.54
	Latency to enter arena [s]	352.50 ± 363.17	419.63 ± 405.42	209.75 ± 293.21	340.88 ± 283.30	133.00 ± 86.52	244.43 ± 240.68
LM	1st Trial Errors [#]	90.13 ± 79.47	45.50 ± 18.27	45.13 ± 21.94	81.38 ± 64.60	84.00 ± 53.88	93.14 ± 43.04
	2nd Trial Errors [#]	18.00 ± 10.95	21.63 ± 9.15	32.25 ± 17.57	91.38 ± 61.65	50.38 ± 50.53	49.86 ± 49.66
	1st Trial Time needed [s]	23.63 ± 22.96	9.25 ± 4.77	10.00 ± 5.61	8.00 ± 4.14	12.25 ± 10.04	15.00 ± 6.88
	2nd Trial Time needed [s]	4.00 ± 4.66	5.88 ± 3.52	7.00 ± 5.42	7.75 ± 5.97	13.00 ± 23.83	8.14 ± 7.54
